# Short-Term Fasting Selectively Influences Impulsivity in Healthy Individuals

**DOI:** 10.3389/fpsyg.2020.01644

**Published:** 2020-07-14

**Authors:** Maxine Howard, Jonathan P. Roiser, Sam J. Gilbert, Paul W. Burgess, Peter Dayan, Lucy Serpell

**Affiliations:** ^1^Department of Clinical, Educational and Health Psychology, University College London, London, United Kingdom; ^2^Institute of Cognitive Neuroscience, University College London, London, United Kingdom; ^3^Gatsby Computational Neuroscience Unit, University College London, London, United Kingdom; ^4^Eating Disorder Service, North East London NHS Foundation Trust, Orchards Health & Family Centre, Essex, United Kingdom

**Keywords:** fasting, short-term starvation, hunger, impulsivity, bulimia nervosa

## Abstract

Previous research has shown that short-term fasting in healthy individuals is associated with changes in risky decision-making. The current experiment was designed to examine the influence of short-term fasting in healthy individuals on four types of impulsivity: reflection impulsivity, risky decision-making, delay aversion, and action inhibition. Participants were tested twice, once when fasted for 20 h, and once when satiated. Participants demonstrated impaired action inhibition when fasted; committing significantly more errors of commission during a food-related Affective Shifting Task. Participants also displayed decreased reflection impulsivity when fasted, opening significantly more boxes during the Information Sampling Task (IST). There were no significant differences in performance between fasted and satiated sessions for risky decision-making or delay aversion. These findings may have implications for understanding eating disorders such as Bulimia Nervosa (BN). Although BN has been characterized as a disorder of poor impulse control, inconsistent findings when comparing individuals with BN and healthy individuals on behavioral measures of impulsivity question this characterization. Since individuals with BN undergo periods of short-term fasting, the inconsistent findings could be due to differences in the levels of satiation of participants. The current results indicate that fasting can selectively influence performance on the IST, a measure of impulsivity previously studied in BN. However, the results from the IST were contrary to the original hypothesis and should be replicated before specific conclusions can be made.

## Introduction

Impulsivity has been defined as behavior that can lead to undesirable consequences, is inappropriate to the circumstance, risky, or ill-considered (Daruna and Barnes, 1993). Impulsivity can be categorized into several subtypes, assessed through self-report and behavioral measures, and is widely implicated in psychiatric illness (Evenden, 1999; Reynolds et al., 2006). Impulsivity is a multifactorial construct that includes several sub-components. There has been extensive research on different models of impulsivity, with researchers concluding that impulsivity is best defined as a combination of several independent yet interacting factors (Evenden, 1999). The following four components of impulsivity were chosen for the current study as they are hypothesized to represent different aspects that may be selectively influenced by a state manipulation, such as fasting. These components are reflection impulsivity, action inhibition, delay aversion, and risky decision-making (Evenden, 1999).

Reflection impulsivity refers to a reluctance to collect and reflect on information before making a decision, and is commonly measured using the Matching Familiar Figures Test (MFFT) (Kagan et al., 1964). Action inhibition has been defined as the failure to inhibit a motor response, and is commonly measured using go/no-go tasks (Murphy et al., 1999). Risky decision-making is the tendency to select a larger, but less likely, versus a smaller, but more likely reward and has been measured in a number of different ways, including the Iowa Gambling Task (IGT) (Bechara et al., 1994; Dunn et al., 2006). The concept of delay aversion has been captured by tasks such as the Temporal Discounting Task that measures the degree to which an individual is driven by immediate gratification vs. the prospect of a delayed reward (Pine et al., 2009).

There has been considerable interest in recent years into the impact of periods of fasting on neurocognitive performance (Benau et al., 2014). Research has demonstrated detrimental effects of fasting on mood, behavior, and cognition (Benau et al., 2014). Such studies have potential implications for understanding the impact of fasting during diets (particularly those which involve intermittent fasting), or religious fasting such as during Ramadan, as well as potentially for eating disorders. In terms of impulsivity, acute starvation has previously been associated with changes in impulsive behavior (Fessler, 2003). In one study, healthy individuals were more risk seeking after fasting for 4 h, compared to when satiated (Levy et al., 2013). However, other studies find healthy individuals to be more risk averse when fasted compared to satiated (Symmonds et al., 2010). The metabolic state of an individual is theorized have an influence on risky decision making by increasing the value of reward, such as food. This can be understood from an evolutionary perspective, as higher risk taking is needed to prevent starvation in conditions of scarce food availability (Symmonds et al., 2010).

The excessive eating and compensatory behaviors observed in bulimia nervosa (BN) have previously been understood in terms of problems of impulse control (Newton et al., 1993). Early research suggested that individuals with BN score higher than healthy individuals on self-report measures of impulsivity (Newton et al., 1993; Fischer et al., 2003; Claes et al., 2006; Myers et al., 2006; Yeomans et al., 2008). However, the ability of one measure to capture all aspects of impulsive behavior have been questioned (Cyders and Coskunpinar, 2011). More recently, studies have used behavioral tasks to measure different facets of impulsivity in BN. In terms of reflection impulsivity, two studies found that individuals with BN were more impulsive on the MFFT (Kaye et al., 1995). However, another study found no difference between BN and healthy controls (Southgate et al., 2008). Studies of action inhibition comparing BN and healthy controls have provided mixed results (Mobbs et al., 2008). A recent meta-analysis concluded that there was stronger evidence for a deficit of action inhibition for disorder-relevant stimuli (food, and body words/images) in BN compared to standard go/no-go tasks (Wu et al., 2013). Four studies using the IGT have shown increased risky decision-making for BN when compared to healthy individuals (Boeka and Lokken, 2006; Liao et al., 2009; Brogan et al., 2010; Chan et al., 2014). However, one study found no differences (Guillaume et al., 2010). In terms of delay aversion, a recent study showed increased temporal discounting in BN (Kekic et al., 2016).

In summary, recent studies using behavioral measures of impulsivity have shown inconsistent results, suggesting that the clinical stereotype of BN as a disorder of poor impulse control may be an oversimplification (Claes et al., 2006). It has been noted that behavioral tasks aimed at the measurement of impulsivity may most effectively characterize state-like, rather than trait-like, contributions that are associated with component cognitive processes such as attention or memory and that may be of particular importance to eating disorders with substantial fluctuations in state (Dougherty et al., 2002; Cyders and Coskunpinar, 2011).

The variable findings in studies examining impulsivity in BN could be accounted for by several factors. Firstly, studies have utilized different tasks, which makes comparisons difficult and limits generalizability (Dunn et al., 2006). Secondly, although researchers have matched healthy individuals and BN groups based on Body Mass Index (BMI), a marker of chronic starvation, short-term eating behaviors are not routinely measured. Individuals with BN may engage in acute starvation (short-term fasting) in order to compensate for over-eating. As mentioned earlier, acute starvation has previously been associated with changes in impulsive behavior (Fessler, 2003; Benau et al., 2014).

Hence, the current study aimed to examine the effect of short-term fasting on performance on well designed and validated tasks measuring four components of impulsivity in healthy individuals, using a within subject, repeated measures design.

In line with the findings that human risk attitudes vary as a function of metabolic state (Symmonds et al., 2010; Levy et al., 2013), and risk seeking behavior in animals increases following fasting (Fessler, 2003), the primary hypothesis was that (1) short-term fasting would increase risky (i.e., low probability) choices during decision-making. Additionally, the effect of short-term fasting on measures of action inhibition, reflection impulsivity, and delay aversion were explored. It was hypothesized that: (2) short-term fasting would be associated with an increase in commission errors on a task of action inhibition; (3) short-term fasting would decrease the amount of information sampled before making a decision on a task of reflection impulsivity; and (4) short-term fasting would decrease the amount of time individuals are willing to wait to receive a reward during a delay aversion task.

## Materials and Methods

### Participants

Power calculation for a repeated measures, within subject ANOVA with a small effect size (0.25) and 90% power conducted in G^∗^Power indicated a required sample size of 30. Thirty-three female participants (mean age = 25 years; *SD* = 8.26; range = 18.5–56) were recruited through the University College London (UCL) subject pool. Eligible participants were female, aged 18–50, and had a BMI > 18.5, with no self-reported history, or current diagnosis, of an eating disorder. Participants were excluded if: they were currently being treated for any serious medical or psychological condition, including diabetes; they had any history of neurological illness or head injury; or were currently pregnant or breastfeeding. The Mini International Neuropsychiatric Interview (MINI), was used to assess any symptoms suggestive of an DSM-IV Axis 1 disorders (Lecrubier et al., 1997). Participants either received course credits or were reimbursed for their time. The research was approved by the University College London Ethics Committee, ref 2337/001. Participants gave written informed consent and a full debrief was provided at the end of the study.

### Procedure

The study used a within-subjects repeated-measures design, assessing behavior under two conditions: once when participants had fasted for 20 h; and once when satiated. The mean time between sessions was 7.2 days (*SD* = 1.7, range = 6–16), with each session lasting 90 min. During the first session participants underwent the MINI (Lecrubier et al., 1997), and completed four behavioral tasks. During the other session participants completed questionnaires and the same behavioral tasks. Task and session order (fasted/satiated) were counterbalanced and randomized. Fasting adherence was assessed using self-reported hunger and blood glucose readings from the distal phalanx area of the index finger using the Freestyle Freedom Lite Blood Glucose Monitoring System, supplied by Abbott Diabetes Care, United Kingdom^1^. All behavioral tasks were administered on a laptop computer, positioned approximately 60 cm from the participant.

### Measures

#### Questionnaires

Participants completed the trait questionnaires once only. These included the Beck Depression Inventory (BDI–II, Beck et al., 1996) a measure of the severity of depressive symptoms; the Eating Disorder Examination Questionnaire-6 (EDEQ-6; Fairburn and Beglin, 1994), to measure ED symptoms; The Impulsive Behavior Scale (UPPS; Whiteside and Lynam, 2001), to measure impulsivity (Mischel et al., 1992; Kirby, 2009; Odum, 2011). Additionally, participants filled in the State-Trait Anxiety Inventory (STAI; Spielberger et al., 1983) and a state measure of hunger at both sessions. This measure consisted of four Likert scales measuring hunger, desire to eat, the amount of food the participant could eat, and fullness. Participants were also asked to rate from *not at all* to *very much so* how much they were experiencing each of the following: dry mouth, stomach aches, anxiety, dizziness, weakness, nausea, thirst, headache, and stomach growling. A composite score was calculated by adding together the four likert ratings associated with the subjective hunger and the nine ratings of physical side effects. A higher score indicated higher levels of self-reported hunger.

### Experimental Tasks

#### Information Sampling Task (Clark et al., 2006) to Measure Reflection Impulsivity

The Information Sampling Task (IST) measures the degree to which participants sample information before making a decision, whilst placing minimal demands on visual processing and working memory. Participants are shown a 5 × 5 matrix of 25 gray boxes and are told that each gray box covers one of two possible colors. Participants must decide which color they think is in the majority, and can click to uncover as many boxes as they wish before deciding. Once opened, boxes remain visible for the remainder of that trial. Correct decisions in the Fixed Win (FW) condition are awarded 100 points, irrespective of number of boxes opened. In the Decreasing Win (DW) condition the number of points to be won decreases by 10 points with every box opened. Therefore in the DW condition participants must tolerate higher uncertainty to win a high number of points as sampling information to a point of high certainty would win few points.

#### Temporal Discounting Task (TDT, Pine et al., 2009) to Measure Delay Aversion

Temporal discounting is the degree to which individuals discount future reward, such as deciding whether to spend in the near future or whether to save for the further future, (Pine et al., 2009). Subjects generally prefer near (spending) to far (saving) reward, consistent with values being discounted in line with the relevant time delay (temporal discounting). The steeper the discounting, the greater the impulsivity. Participants were asked to choose between two serially presented options of differing magnitude ranging from a monetary value of 1 to €100, and a time delay of one week to one year. The rate at which future reward are discounted (*k*) is used as a measure of delay aversion. Participants with a greater discount rate devalue future reward more quickly. Participants were told that one of the options they chose would be randomly selected and paid for on a pre-paid card with a timed activation date, as used in the original study. However, they were debriefed at the end of the task and no payment was made. The task also contained 20 trials in which one of the choices presented was always larger and available sooner. These “catch” trials were used to determine the subject was paying attention to, and understood the task.

#### Choice x Risk Task (CRT, Rogers et al., 2003) to Measure Risky Decision Making

The Choice × Risk task is used to investigate three factors thought to affect decision-making: the magnitude of expected gains (reward), the magnitude of expected losses (punishment) and the probabilities of each. On each trial participants were required to choose between two gambles, represented as two bars simultaneously presented on the screen. The amount the bar is filled represents the probability of winning, while wins and losses are displayed numerically at the top and bottom of each bar in green and red text, respectively. Participants complete four games, consisting of 20 trials presented in a pseudorandom order. There are eight repetitions of each of 10 trial types, including “gain only” and “loss only” trials. Participants were given 100 points at the beginning of each game and instructed to win as many points as possible. After each trial feedback was given on performance and an updated score was displayed for 2 s.

Standard trial types always contained a control gamble (50/50 chance of winning 10 points) and an experimental gamble. The experimental gamble varies in the probability of winning to be either high or low (75 vs. 25), expected gains are either large or small (80 vs. 20 points) and expected losses either large or small (80 vs. 20 points), producing eight trial types. The other two trial types, “gain only” and “loss only” were used to estimate risk-aversion when choosing between losses, and risk-seeking when choosing between gains. In a “gains only” trial, two options with the same expected value are presented. For example, if participants more frequently choose a 100% chance of a gain of €20 when compared to a 50% chance of gaining €40, they would be exhibiting risk-aversion for gains. Similarly, in a “loss only” trial, two options of equal expected value are presented, such as a 50% chance of a €40 loss, compared to a 100% chance of a €20 loss. If participants are more likely to choose the 50% chance of a €40 loss, they would be exhibiting risk-seeking for losses.

#### Affective Shifting Task (AST, Modified From Murphy et al., 1999) to Measure Action Inhibition

The AST is a measure of motor inhibitory control. Subjects see pictures from two classes - target and distractor - presented rapidly, one at a time in the center of the screen. They have to respond to target stimuli by depressing the space bar (go) as quickly as possible, whilst inhibiting responses to distractor stimuli (no-go). The time taken to respond to targets (RTs), failures to respond (omissions), and incorrect responses (commission errors) are recorded, with the latter providing a measure of motor inhibition.

Stimuli were pictures of food (F) or household items (H) taken from an existing database designed for neuropsychological studies of Anorexia Nervosa (AN) (Uher et al., 2004). Instructions at the beginning of each block indicated which stimulus type to respond to. Each stimulus was presented for 300 ms with an inter-trial interval of 900 ms. A 500 ms/450 Hz tone sounded for each error of commission, but not for omissions. There were 10 blocks (2 practice blocks) with 18 stimuli presented in each block, arranged in either of the following orders: FFHHFFHHFF, HHFFHHFFHH. This order means that four blocks were “shift” blocks, in which participants had to respond to stimuli that were previously distractors, and inhibit responding to previous targets. In the “non-shift” blocks participants had to continue responding to the same targets and inhibiting responses to the same distractors as in the immediately previous block. Note that this was the only one of the included tasks which incorporated food stimuli.

### Statistical Analysis

All statistical analyses were performed using SPSS 21 (IBM SPSS, 2010, Chicago, IL, United States). Two tailed statistical significance was determined as *p* < 0.05. Descriptive statistics (mean and standard deviations) were calculated for all demographic and questionnaire scores.

#### Information Sampling Task

To investigate the effect of fasting on the amount of information sampled during the IST, the dependent variable, average number of boxes opened before making a decision, was entered into a multivariate analysis. A mixed model ANOVA with the within-subject factors of Session (fasted, satiated), Condition (Fixed Win, Decreasing Win) and the between-subject factor of Order (FW-DW, DW-FW) was conducted separately on the primary outcome of average boxes opened, and the secondary outcome of errors. Any significant interactions were then explored with Bonferroni corrections applied.

#### Temporal Discounting Task

Impulsive choice was calculated as the number of sooner options chosen by each participant, for each trial, separately for the fasted and satiated sessions. A pairwise comparison was used to examine any differences across fasted and satiated sessions.

Maximum likelihood estimation was used in order to calculate the maximum likelihood parameters for the discount rate (*k*), and utility concavity (*r*). For each of the 220 choices for each participant a Bernoulli likelihood (based on the sigmoid of the difference in discounted value) was calculated for the chosen option). Likelihood maximization proceeded via optimization functions in MATLAB (The MathWorks Inc., Natick, MA, United States). See Pine et al. (2009) for further information and methods. Pairwise comparisons were run to examine any differences in the discount rate (*k*), or utility concavity (*r*), between fasted and satiated sessions.

#### Choice × Risk Task

To examine the effect of fasting on risky decision-making, multivariate analysis was conducted on the number of times participants choose the experimental, over the control, gamble (proportionate choice) and the mean deliberation times associated with these choices. The data was proportional and was therefore arcsine transformed prior to statistical analysis in line with the analysis method described by Rogers, (Rogers et al., 1999). However, all values presented in tables are untransformed scores, for ease of interpretation.

The proportionate choices were analyzed using a within subjects repeated measures 2 × 2 × 2 × 2 ANOVA with the factors of session (fast vs. satiated), probability (high vs. low), expected gains (large vs. small), and expected losses (large vs. small). This ANOVA was then repeated with mean deliberation times (ms) as the dependent variable.

The “gains only” and “losses only” trials were analyzed using a within subjects repeated measures 2 × 2 ANOVA with session (fast vs. satiated), and trial type (“gains only” vs. “losses only”). Analysis was conducted on both proportion and deliberation times separately.

#### Affective Shifting Task

To determine the effect of fasting on performance during the AST, multivariate analyses were conducted separately on reaction times (ms), errors of commission, and errors of omission using a 2 × 2 × 2 repeated measures ANOVA with Stimuli (food, household); Condition (shift, non-shift); and Session (fast, satiated) entered as within-subject factors. Any significant interactions were then explored and the Bonferroni correction was applied.

## Results

Demographic characteristics and questionnaire scores are displayed in Table 1. All participants were included in analysis as none were identified as having a current or lifetime history of psychiatric disorder, as assessed using the MINI.

**TABLE 1:** Means and standard deviations for demographic variables and trait measures (*n* = 33).

Demographic Variables	Mean ± *SD*
Age (years)	25.428.26
Body Mass Index (BMI)	21.653.22
**Trait Measures**	
*UPPS-P subscales*	
Negative Urgency	274.97
Lack of Premeditation	22.885.09
Lack of Perseverance	18.553.85
Sensation Seeking	35.396.66
Positive Urgency	26.457.04
BDI	5.154.87
EDE-Q	1.351.13
STAI	39.309.96

*UPPS-P, the Impulsive Behavior Scale; BDI, Beck Depression Inventory; EDE-Q, Eating Disorder Examination Questionnaire-6; STAI, State-Trait Anxiety Inventory.*

### Physiological Analysis

#### Blood Glucose

Pairwise comparisons revealed a significant difference for blood glucose levels (mmol/L) between fasting and satiated sessions *t*(32) = −5.07, *p* < 0.001. Blood glucose levels in the fasted session (*M* = 4.06, *SD* = 0.51) were lower than in the satiated session (*M* = 4.90, *SD* = 0.871).

### Information Sampling Task

Accuracy scores for identifying the correct box color were examined and two participants with accuracy scores lower than 60% were excluded from further analysis, in line with the original study (Clark et al., 2006).

### Boxes Opened

There was a significant main effect of Session [*F*(1,28) = 9.72, *p* = 0.004], a significant main effect of Condition [*F*(1,28) = 76.16, *p* < 0.001] and a significant Session x Condition interaction [*F*(1,28) = 4.49, *p* < 0.05]. There was no significant effect of Condition Order for the fasting [*F*(1,28) = 0.008, *p* = 0.928] or satiated Session [*F*(1,28) = 0.284, *p* = 0.599]. Pairwise comparisons revealed that participants opened significantly fewer boxes in the DW condition, compared to FW for both fasting *t*(30) = 7.86, *p* < 0.001 and satiated *t*(30 = 6.78, *p* < 0.001) sessions, see Table 2 for mean scores.

**TABLE 2:** Mean difference and standard deviation (±) scores across fasted and satiated sessions.

		Boxes opened	Errors
Fasted	DW condition	10.414.08	1.901.33
	FW condition	17.074.45	0.710.90
Satiated	DW condition	9.793.72	2.101.42
	FW condition	13.735.05	1.291.22

*Post hoc* analysis revealed a significant difference between sessions in the FW condition *t*(30) = 3.81, *p* = 0.001 but not the DW condition *t*(30) = 1.41, *p* = 0.168. During the FW condition participants opened more boxes before making a decision, when fasted (*M* = 17.07, *SD* = 4.45) compared to when satiated (*M* = 13.73, *SD* = 5.05).

#### Errors

Analysis of error data using a mixed model ANOVA showed a significant main effect of Session [*F*(1,28) = 5.75, *p* < 0.05], and a significant main effect of Condition[*F*(1,28) = 22.21, *p* < 0.001]. The Session × Condition interaction was not significant [*F*(1,28) = 0.744, *p* = 0.396]. Participants made a higher number of errors during the satiated session, and more errors during the DW condition, see Table 2 for mean scores and standard deviations.

### Temporal Discounting Task

Two participants scored under 80% on the catch trials across both sessions and were therefore excluded from further analysis. All other participants had high accuracy (mean = 19.15) on the catch trials (out of a possible 20). Participants varied on the number of trials in which the sooner option was chosen, ranging from 2 to 184, out of a possible 200 trials. The model of best fit from Pine et al. (2009) showed that participants discounted the value of future reward (mean fasted *k* = 0.06, SD = 0.68; mean satiated *k* = 0.07, SD = 0.066) and demonstrated a concave utility function (mean fasted *r* = 0.0213, SD = 0.03609; mean satiated *r* = 0.0140, SD = 0.02830). However, the discount rate *t*(30) = −0.521, *p* = 0.606 and concave utility *t*(30) = 1.438, *p* = 0.161 were not significantly different between fasted and satiated sessions. The impulsive choices made did not differ across session *t*(30) = −0.327, *p* = 0.746.

### Choice × Risk Task

Data from three participants were missing for the Choice x Risk Task due to a recording error; therefore 30 participants were included in the following analyses.

#### Probability, Wins, and Losses

##### Proportionate choice

There was no main effect of Session (fasted, satiated) on the proportion of times that participants chose the “experimental” gamble over the “control” gamble [*F*(1,29) = 0.22, *p* = 0.643]. However, participants chose the “experimental” gamble significantly more often when the probability of winning was high compared to when it was low, [*F*(1,29) = 204.73, *p* < 0.001], significantly less often when the expected losses were large compared to small [*F*(1,29) = 32.95, *p* < 0.001], and significantly more often when the expected gains were large compared to when they were small [*F*(1,29) = 28.30, *p* < 0.001]. However, there was no significant interaction that involved Session (fasted vs. satiated).

##### Deliberation times

There was no main effect of Session [*F*(1,29) = 1.41, *p* = 0.26], Probability [*F*(1,29) = 1.90, *p* = 0.18], or Expected Gains [*F*(1,29) = 0.34, *p* = 0.57], but a significant main effect of Expected Losses [*F*(1,29) = 8.72, *p* < 0.01]. Participants took longer to choose when the “experimental” gamble was associated with large expected losses compared to small losses. Means and standard deviations are presented in Table 3. There was no significant interaction that involved Session (fasted vs. satiated).

**TABLE 3:** Proportion of choices of the “experimental” over the “control” gamble for the probability of winning, expected losses and gains across fasted and satiated sessions.

	Probability of winning on the “experimental” gamble		Levels of expected losses on “experimental” gamble		Levels of expected gains on “experimental” gamble	
Group	High	Low	Large	Small	Large	Small
Fasted	0.77 ± 0.33	0.18 ± 0.18	0.45 ± 0.25	0.62 ± 0.21	0.59 ± 0.23	0.48 ± 0.20
Satiated	0.78 ± 0.30	0.14 ± 0.13	0.46 ± 0.22	0.61 ± 0.18	0.58 ± 0.20	0.48 ± 0.18

#### “Gains Only” vs. “Losses Only” Trials

##### Proportionate choice

Participants chose the guaranteed options significantly more often on the “gains only” trials compared to the “losses only” trials [*F*(1,29) = 83.07, *p* < 0.001]. Overall choice was unaffected by Session [*F*(1,29) = 0.41, *p* = 0.53] and the interaction between session and trial type was non-significant [*F*(1,29) = 0.85, *p* = 0.77].

##### Deliberation times

Table 4 shows mean deliberation times on for high and low probabilities of winning and large and small losses for fasted and satiated conditions. Participants were significantly faster to choose on the “gains only” trials compared to the “losses only” trials [*F*(1,29) = 12.34, *p* = 0.001]. Reaction times were unaffected by Session [*F*(1,29) = 1.11, *p* = 0.30] and the interaction between session and trial type was non-significant [*F*(1,29) = 0.314, *p* = 0.58].

**TABLE 4:** Mean deliberation times (ms) and standard deviation scores for probability of winning, expected losses and gains across fasted and satiated sessions.

	Probability of winning on the “experimental” gamble		Levels of expected losses on “experimental” gamble		Levels of expected gains on “experimental” gamble	
Group	High	Low	Large	Small	Large	Small
Fasted	1637 ± 729	1674 ± 642	1733 ± 740	1577 ± 609	1655 ± 650	1656 ± 683
Satiated	1811 ± 1008	1954 ± 1180	1936 ± 1140	1829 ± 1028	1902 ± 1149	1862 ± 1026

### Affective Shifting Task

#### Reaction Time

There was a significant main effect of Stimuli [*F*(1,32) = 15.26, *p* < 0.001], and Condition *F*(1,32) = 5.38, *p* < 0.05, but no significant effect of Session [*F*(1,32) = 0.25, *p* = 0.617]. There was no significant interaction between: Session and Condition [*F*(1,32) = 1.76, *p* = 0.194]; Session and Stimuli (*F*(1,32) = 1.34, *p* = 0.26); Condition and Stimuli [*F*(1,32) = 0.48, *p* = 0.49]; or between Session, Condition and Stimuli [*F*(1,32) = 0.08, *p* = 0.78].

Overall, reaction times (RTs) for food stimuli were shorter (*M* = 462.65, *SD* = 57.89) than for household items (*M* = 482.02, *SD* = 56.70). Non-shift trials also had shorter RTs (*M* = 468.44, *SD* = 57.55), compared to shift trials (*M* = 476.24, *SD* = 57.04).

#### Errors of Commission

There was a significant main effect of Session [*F*(1,32) = 5.39, *p* < 0.05] but not of Stimuli [*F*(1,32) = 0.15, *p* = 0.69]. There was also a significant main effect of Condition [*F*(1,32) = 43.5, *p* < 0.001]. The interaction between Session and Stimuli was not significant [*F*(1,32) = 2.88, *p* = 0.10], nor was the interaction between Session and Condition [*F*(1,32) = 0.27, *p* = 0.610], or Stimuli by Condition [*F*(1,32) = 0.16, *p* = 0.695]. However there was a significant interaction between Session, Stimuli, and Condition [*F*(1,32) = 4.82, *p* < 0.05].

More commission errors were made during the fasted session (*M* = 1.55, *SD* = 0.89), than the satiated session, (*M* = 1.19, *SD* = 0.82). Participants also made a higher number of commission errors for shift (*M* = 1.41, *SD* = 1.02), compared to non-shift conditions (*M* = 0.14, *SD* = 0.81).

**FIGURE 1:**
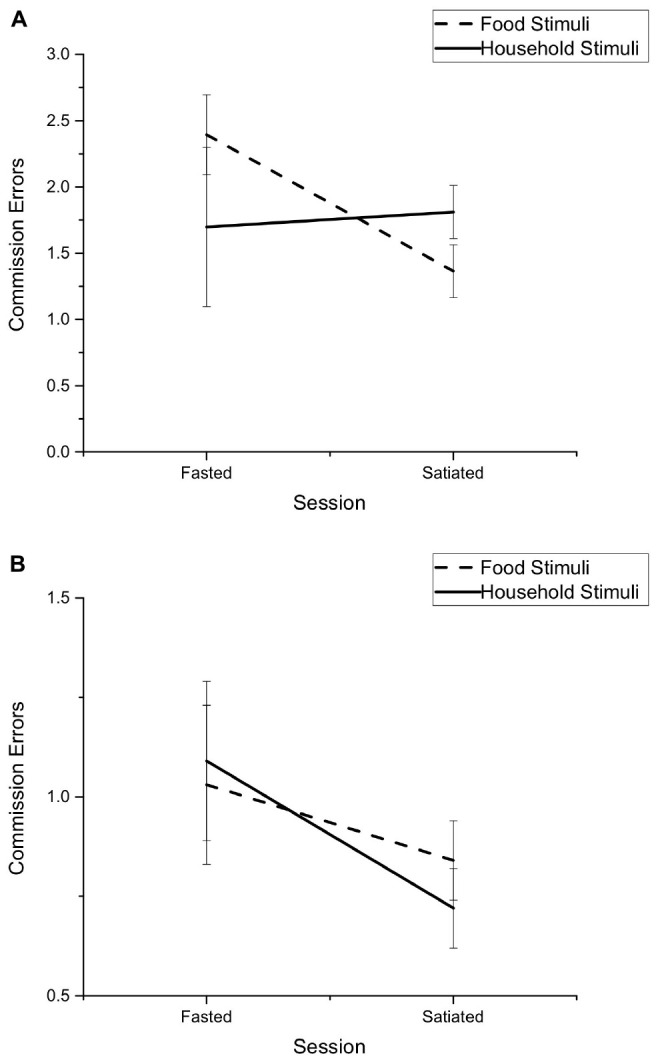
Mean number of commission errors made during the Affective Shifting Task for food and household stimuli across fasted and satiated sessions. Presented separately for **(A)** Non-shift condition, **(B)** Shift condition. Error bars represent standard error.

Bonferroni *post hoc* comparisons to explore the Session by Stimuli by Condition interaction showed that there was no difference in the number of commission errors made toward household items between fasted and satiated sessions, for either shift (*p* = 0.33) or non-shift (*p* = 0.23) blocks. There was also no difference in commission errors toward food stimuli for fasted or satiated sessions during the non-shift block (*p* = 0.44). However, there was a significant difference in the number of commission errors in response to food stimuli during the shift blocks (*p* < 0.05). There was a higher number of commission errors in response to food stimulus during fasted (*M* = 2.39, *SD* = 2.21) compared to satiated sessions (*M* = 1.36, *SD* = 1.48), see Figure 1.

#### Errors of Omission

There was no main effect of Session *F*(1,32) = 0.62, *p* = 0.44 or Stimuli *F*(1,32) = 0.005, *p* = 0.95. However, there was a significant main effect of Condition *F*(1,32) = 6.17, *p* < 0.05. The interaction between Session and Stimuli was not significant, *F*(1,32) = 0.88, *p* = 0.36, nor was the interaction between Stimuli and Condition *F*(1,32) = 0.25, *p* = 0.62, nor the interaction between Session, Stimuli, and Condition *F*(1,32) = 0.42, *p* = 0.517. There was a significant interaction between Session and Condition *F*(1,32) = 7.52, *p* < 0.05. Participants made a higher number of errors of omission during shift blocks (*M* = 1.06, *SD* = 0.90), compared to non-shift blocks (*M* = 0.77, *SD* = 0.87). The Session by Condition interaction was explored using Bonferroni adjusted comparisons and revealed that participants made more errors of omission during shift blocks when satiated (*p* < 0.05). However, there was no difference in omission errors between shift and non-shift blocks when fasted (*p* = 0.44).

#### Relationship Between Self-Report and Behavioral Measures

Change scores between satiated and fasted sessions were calculated for the commission errors made during the AST, and for the number of boxes opened during the FW condition of the IST. Change scores for the state self-report measures were also calculated (STAI, blood glucose, and hunger). Correlations between these variables were then calculated. However, there was no significant correlation between the self-report measures and difference scores for the IST and AST. See Table 5. Mean centered age was added as a covariate in all models to examine any association between age and performance on the cognitive tasks, in addition to any interaction with fasting. There was neither significant main effect of age nor interaction with fasting across any of the tasks.

**TABLE 5:** Pearson correlations between the IST and AST difference scores (satiated minus fasted) and state changes in Anxiety, Blood Glucose and Hunger.

	Difference between Satiated and Fasted Sessions	
	IST Boxes Opened FW Condition	AST Commission Errors
**Demographic Variables**		
Age (years)	−0.12	−0.10
Body Mass Index (BMI)	−0.28	−0.07
**Trait Measures**		
*UPPS-P subscale*s		
Negative Urgency	−0.07	0.03
Lack of Premeditation	0.24	−0.06
Lack of Perseverance	−0.12	−0.17
Sensation Seeking	−0.06	−0.02
Positive Urgency	−0.17	0.04
BDI	0.09	0.00
EDE-Q	−0.06	−0.12
Trait-STAI	0.01	−0.19
**State Measures (Difference Scores)**		
State-STAI	0.16	0.04
Blood Glucose	0.14	0.16
Hunger	0.17	−0.00

*All correlations were non-significant, *P* > 0.05. IST, Information Sampling Task; AST, Affective Shifting Task; UPPS-P, the Impulsive Behavior Scale; BDI, Beck Depression Inventory; EDE-Q, Eating Disorder Examination Questionnaire-6; STAI, State-Trait Anxiety Inventory.*

## Discussion

This study aimed to examine the effect of short-term fasting on tasks measuring four components of impulsivity. Results showed that, contrary to expectations, participants took longer and opened more boxes in the Fixed Win (FW) condition of the Information Sampling Task (IST), a measure of reflection impulsivity, in the fasted compared to the satiated state. Additionally, short-term fasting was associated with more commission errors during the Affective Shifting Task (AST), indicative of a deficit in action inhibition. When fasted, participants made significantly more errors of commission for food compared to household stimuli during shift blocks. There was no difference between fasted and satiated sessions on the impulsive choices made during the Temporal Discounting Task, or in risky decision-making during the Choice × Risk Task.

Participants opened more boxes and made fewer errors in the Fixed Win (FW) condition of the IST when fasted, indicating a decrease in reflection impulsivity. However, there were no fasted/satiated differences for the Decreasing Win (DW) condition. This suggests that the two conditions were differentially affected by fasting. During the DW condition participants were told that with every box opened, the number of points to be won decreases, hence there is a cost to opening more boxes. However, during the FW condition participants are told that they can open as many boxes as they wish, with no decrease in winnings. An adaptive strategy would be to open all boxes to guarantee a win. However, participants typically guess before all of the boxes have been opened (Clark et al., 2006).

The results of the IST were contrary to the hypothesis that short-term fasting would be associated with increased reflection impulsivity. The decreased reflection impulsivity displayed during the fasted session could be due to a number of different factors. Firstly, the ability to flexibly shift attention between decision making (deciding which box color is in the majority), and the action of box opening could be affected by fasting, causing the “repetitive” box opening during the FW condition. This is unlike the DW condition, in which participants are cued by the decreasing points to shift from opening boxes to make a decision about which color is in the majority. Set-shifting is the process of changing, or switching, between responding to different tasks, rules, or mental sets (Uher et al., 2004), and has been extensively studied in Eating Disorders (ED), (Uher et al., 2004). Recent research (Benau et al., 2014) has demonstrated that fasting affects set-shifting, particularly with cue-induced craving (Piech et al., 2009; Benau et al., 2014), and that 18 h of fasting exacerbates set-shifting difficulties on a rule change task (Bolton et al., 2014). Although this type of short-term fasting in a healthy population is not identical to the patterns of food restriction and chronic or intermittent fasting seen in EDs, it could explain, in part, why participants opened more boxes in the FW condition of the IST when fasted.

Secondly, participants in the fasted session may have become fixated on the detail of opening each box individually and were unable to stand back to see the “whole picture” to make a decision. The term central coherence is used to refer to the ability to combine information into the “bigger picture” rather than focusing only on the finer detail. An impairment in central coherence has been shown in individuals with ED’s (Lopez et al., 2008) and fasted participants (Pender et al., 2014). However, an impairment in central coherence may not have occurred in the DW condition as participants may have been cued into making a decision by the decreasing points. Alternately, participants in the fasted condition may have been more indecisive as a consequence of fasting, needing to reach a higher criterion of certainty before making a decision.

However, it is not possible to determine the contribution of either of these explanations from the current experiment. Therefore the results require further investigation and replication to understand the mechanisms underpinning the effect of decreased reflection impulsivity on the IST.

Results from the current study indicate that short-term fasting did not affect delay aversion. Participants in the fasted condition did not choose to delay the receipt of a monetary reward any less than when satiated. However, participants may have been less susceptible to the fasting manipulation as the hypothetical on-screen choices are viewed as more distant, compared to immediate presentation, and are more objectively assessed (Rachlin, 2009). The degree to which an individual discounts future reward has also been described as a trait characteristic (Odum, 2011), and is stable over time (Mischel et al., 1992; Kirby, 2009). Therefore manipulating the state of the individual (fasting) may not influence an established trait discount rate toward monetary reward. Research has shown pharmacological effects on “trait” measures of impulsivity, such as the Choice x Risk task (Rogers et al., 2003) using a within subjects design.

Participants also showed no difference between fasted and satiated sessions for the different probabilities of winning, different magnitudes of expected losses, and expected gains on the Choice x Risk Task. This indicates that risky decision-making was not influenced by short-term fasting. This finding is in contrast to previous research that found increased risky decision-making for food, water, and money following 4 h of food and water deprivation (Levy et al., 2013). However, this could be related to differences in the salience of the reward as participants in the current study received points rather than food, water, or money, which may be differentially affected by fasting. Additionally, exploratory analysis of fasted state on risk preferences in Levy et al. (2013) study revealed a small effect (5% change) that appeared to be related to the baseline characteristics of the included sample.

Another study demonstrated that risky decision-making decreased when fasted participants were provided with a meal to reach satiation. However, this study involved exclusively male participants (Symmonds et al., 2010), whereas, the participants in the current study were all female. Hence, gender differences might account for the inconsistent results, especially when males and females have been shown to respond to fasting differently (Uher et al., 2006). Furthermore, the effect on risky decision-making in the previous study was only significant immediately after a satiated meal but not 1 h later (Symmonds et al., 2010). This appears to be in line with the current lack of effect of fasting given that participants in the current study were told to eat normally prior to the satiated session, and were not provided with food during task completion which took between 30 and 60 min.

Participants exhibited more errors of commission for food stimuli during the AST when fasting compared to when satiated, indicating a deficit of action inhibition. However, there were no differences in response times between fasted and satiated sessions. The increased number of errors of commission in the fasted condition indicated decreased action inhibition. Higher errors of commission, or decreased action inhibition, in BN compared to healthy individuals have previously been interpreted as indicative of greater impulsivity (Mobbs et al., 2008). Participants made significantly more commission errors when fasted during the more difficult shift blocks for food compared to household stimuli. This difference was not present in the non-shift blocks. This result could indicate that participants are less able to control motor impulsivity during a more demanding task, and toward food stimuli when fasted.

Therefore the current findings suggest that short-term fasting may be an important consideration when examining differences in action inhibition between controls and BN. If individuals with BN undergo periods of short-term fasting, and have a similar response to healthy individuals in the current study, then the increased commission errors in BN could be attributed to fasted state, rather than reflecting an impulsive neurocognitive profile, or trait. It is important to disentangle the contribution of short-term fasting to impulsivity seen in BN so that treatments that focus on reducing impulsivity such as Dialectical Behavior Therapy can be appropriately informed and targeted.

A limitation of the current experiment is the inability to address whether the differences found between fasted and satiated sessions is due to the primary effect of lowered blood glucose on brain function, or the secondary effect of hunger (induced through fasting) influencing motivation, or fatigue. Previous research indicates that changes in cognition can be independent of blood glucose, and may be mediated by other factors (Pollitt et al., 1983), and could be controlled by homeostatic mechanisms not assessed in the current study (Cryer, 1981). Green et al. (1995) have previously found that although there was a significant difference between self-reported hunger for fasted and satiated sessions, task performance was not affected. This indicates that subjective measures of hunger may not always relate to differences in task performance. The tasks in the current study for which there were non-significant findings may not have sensitive enough to detect subtle differences in performance that could occur as a result of fasting (Green et al., 1995). Further research is needed in order to examine the role of subjective hunger on cognition and to separate out the influence of primary and secondary effects of fasting on cognitive performance. It should also be noted that this type of laboratory task is sensitive to cognitive processes other than impulsivity such as attention and memory (Cyders and Coskunpinar, 2011), performance on which has actually been shown to improve with intermittent fasting (Mattson and de Cabo, 2020).

Previous research has shown that there is an influence of age on impulsivity (Steinberg et al., 2008), particularly in early adolescence. However, we found no significant effect of age on task performance or fasting manipulation. This could have been due to the limited age range of our participants, reducing the power to detect differences. It should be considered a further limitation.

The fasting manipulation might not have increased the value of a monetary reward, but instead increased the value of a food reward. Previous studies have demonstrated that nicotine deprivation can lead to a steeper discounting rate for cigarettes, but not monetary reward (Mitchell, 2004). This demonstrates that state manipulations can have differential effects on the impulsive choices made in response to different reward. The present findings are therefore only applicable to monetary reward, and future studies should investigate food reward using this paradigm. This could also account for the non-significant findings during the delay aversion and risky-decision making task, which used monetary values as reward. However, the present results show that general delay aversion toward money did not differ as a function of fasting. Including food stimuli during the temporal discounting task could make the results difficult to interpret. It might be hard to separate impulsiveness toward food items as a result of fasting from the increased value of food items caused by food deprivation.

The current study examines differences between fasted and satiated healthy individuals with no known history of an eating disorder. Research has shown that individuals can habituate to periods of short-term starvation and may be able to tolerate changes in cognition over repeated exposure to a fasted state (Mattson and de Cabo, 2020). Therefore individuals with BN, who undergo cycles of restriction, may respond differently when compared to healthy individuals.

It is clear that further studies need to be conducted in order to better understand the effect of short-term fasting in healthy participants. Research should continue to investigate the most appropriate design in which to examine the role of short-term fasting on cognitive performance. In the meantime, caution should be used when interpreting findings from ED participants, particularly BN, as indicative of trait differences in cognitive performance due to the influence of fasted state on these measures.

## Data Availability Statement

The datasets generated for this study are available on request to the corresponding author.

## Ethics Statement

The studies involving human participants were reviewed and approved by the UCL PALS Ethics Committee. The patients/participants provided their written informed consent to participate in this study.

## Author Contributions

MH: project administration, conceptualization, methodology, investigation, formal analysis, and writing – original draft. JR: conceptualization, supervision, and writing – review and editing. SG: formal analysis and writing – review and editing. PB: writing – review and editing. PD: methodology, resources, and writing – review and editing. LS: project administration, supervision, formal analysis, and writing – review and editing. All authors contributed to the article and approved the submitted version.

## Conflict of Interest

The authors declare that the research was conducted in the absence of any commercial or financial relationships that could be construed as a potential conflict of interest.

## Footnotes

1 www.abbottdiabetescare.co.uk
